# Understanding the challenges and gaps in community engagement interventions for COVID-19 prevention strategies in Rohingya refugees: a qualitative study with frontline workers and community representatives

**DOI:** 10.3389/fpubh.2023.1169050

**Published:** 2023-08-03

**Authors:** Charls Erik Halder, Md Abeed Hasan, Yussuf Mohamed Mohamud, Marsela Nyawara, James Charles Okello, Md Nahid Mizan, Md Abu Sayum, Ahmed Hossain

**Affiliations:** ^1^Migration Health Division, International Organization for Migration, Cox’s Bazar, Bangladesh; ^2^College of Health Sciences, University of Sharjah, Sharjah, United Arab Emirates; ^3^Global Health Institute, North South University, Dhaka, Bangladesh

**Keywords:** COVID-19, Rohingya, knowledge, attitude and practice, community health worker, Cox’s Bazar, risk communication, community engagement

## Abstract

**Background:**

Rohingya refugees in Bangladesh are vulnerable to infectious diseases such as COVID-19 due to the crowded living conditions with fragile shelters, and limited water, sanitation and hygiene facilities and practices. While risk communication and community engagement (RCCE) is the cornerstone of outbreak control, there is limited evidence available on the effectiveness of the RCCE strategies in this setting.

**Objectives:**

The goal of this study is to evaluate the effectiveness of RCCE strategies and to explore the challenges and community recommendations in relation to COVID-19 preventive measures in the context of Rohingya refugee camps in Bangladesh.

**Materials and methods:**

It was a qualitative study. Methods used were (a) observation of RCCE intervention by 3 clinical supervisors accompanying 25 Community Health Workers (CHWs) and (b) 5 focus group discussions engaging 60 community representatives. Data were analyzed using a thematic analysis approach, separately for observation and focus group discussions.

**Results:**

The study identified a number of good practices of RCCE, including selecting CHWs from the local community, engaging female CHWs, using local dialect, and collaborating with community/religious leaders. Certain good practices need scaling up, such as utilization of multiple communication methods and interpersonal communication skills. Some areas need improvement, such as CHWs being overburdened with multiple tasks, less effort to active listening, repeated delivery of same messages, inadequate linkage to culture, context, and resources, and less effort to empower the community. Engaging the community, five critical themes were identified in relation to poor COVID-19 preventive practices: culture, religion, and language; local context and resources; community trust and interaction with aid workers; communication methods; and gender and social inclusion. Religious misinterpretation, cultural barriers, physical barriers, lack of resources, breach of trust between the community and aid workers, inconsistent/complex messages, lack of gender and social inclusion, and stigmatization are among some key factors. Some key actions were recommended to improve COVID-19 RCCE strategy.

**Conclusion:**

We urge the RCCE partners to make use of the findings and recommendations to develop a robust RCCE strategy relevant to local culture and context, responsive to people’s concerns and needs, and inclusive of gender, age and social vulnerabilities.

## Introduction

Following the massive displacement from Myanmar in 2017, about 883,600 Rohingya refugees are currently living in 34 overcrowded camps in Ukhiya and Teknaf Upazilas of Cox’s Bazar (1). This was preceded by decades of influxes driven by systematic discrimination and deliberate violence against the Rohingya community (2). The refugees are especially vulnerable to natural and man-made disasters, including outbreaks of infectious diseases since they live in crowded bamboo-made settlements on hilly slopes and basins with limited access to essential livelihood and entitlements (3). The infectious disease epidemics are predisposed by the crowded living conditions with fragile shelters, a lack of adequate water, sanitation, and hygiene (WASH) facilities and practices, and intense monsoons in the refugee camps and surrounding host community (4). Since 2017, the Rohingya camps have experienced epidemic or upsurge of a number of infectious diseases, including diphtheria, measles, acute watery diarrhea, and dengue (4). COVID-19 was a new threat to this community that appeared to be superimposed on the existing susceptibility of the community to different disease outbreaks. As a cluster of viral pneumonia, the disease was first reported in Wuhan in December 2019 and since has spread widely over the world, with more than 500 million confirmed cases and 6 million fatalities reported in 200 countries (5, 6). World Health Organization (WHO) declared COVID-19 outbreak as a global pandemic on March 11, 2020 (7). The first confirmed case of COVID-19 was detected in Bangladesh on March 8, 2020, and the first confirmed case from Rohingya refugee camps was reported in the month of May of the same year (8, 9). As of the June 30, 2022, there were a total of 103,352 tests conducted in the refugee camps, which resulted in the confirmation of 14,731 instances of COVID-19, along with 42 fatalities (10).

There has been a number of public health recommendations issued for the prevention and control of COVID-19, which includes social distancing, use of mask, hand washing, cough etiquette getting vaccinated, staying home while unwell, and seeking medical attention when necessary (11, 12). Public health and social measures remained the most essential instrument for preventing the spread of disease before vaccines became widely available to the general population (13). Therefore, risk communication and community engagement (RCCE) was considered as one of the primary pillars of the COVID-19 response strategy (13).

The International Organization for Migration (IOM) carried out a wide range of RCCE interventions in Cox’s Bazar aiming to reduce the COVID-19 disease transmission through strengthening the capacity of the community to practice public health measures. This included household visits and community meetings facilitated by the community health workers (CHWs); the production and dissemination of information, education and communication materials (e.g., audio-visual clips and printed materials); social advocacy conducted by social leaders and community groups; and “go and see visits” to the service sites. The majority of the communication, both its messages and its contents, was based on materials generated by the Communication with Community (CWC) working group.

The successful implementation of public health measures is largely dependent on what people know about those measures (knowledge), how they think or believe in those measures (attitude), and how they do or experience those measures (practice) (14). Prior to this qualitative study, a quantitative study was carried out by the same study team to assess the level of knowledge, attitude and practice of COVID-19 preventive measures among the community following the RCCE interventions carried out (15). It was cross-sectional study, 500 Rohingya individuals were surveyed using a structured questionnaire. The study found that the mean scores for knowledge, attitude and practice were, respectively, 9.93 (out of 14), 7.55, respectively, (out of 11) and 2.71 (out of 7) indicating that the Rohingya refugee community in Cox’s Bazar had improved knowledge and attitude toward COVID-19 preventive measures, however, the practice level of these measures remained low compared to the knowledge and positive attitude (15). Also, different forums and reports have highlighted the issue of noncompliance among the population with COVID-19 measures; however, there is no evidence as to why the public health measures are not accepted or practiced by the community and/or how the issue can be addressed using local knowledge and resources. Moreover, there is no evidence available on how effective the current communication and community engagement approaches are in terms of inter-activeness, acceptability and comprehensibility. This assessment was carried out through the active involvement of the community and frontline volunteers from November 2021 to January 2022 in order to address these gaps in information and evidence on the COVID-19 practice and RCCE strategy. The findings of the study could support the development of a robust strategy on RCCE for the ongoing pandemic as well as future outbreaks of infectious diseases.

## Materials and methods

### Study area

The research was conducted in Rohingya refugee settlements in Cox’s Bazar, Bangladesh, where IOM Health implemented community health interventions. In Cox’s Bazar refugee camps, there are around 883,600 Rohingya people.

### Study design and participant selection

It was a qualitative study, which used phenomenological approaches. The study was conducted followed by a quantitative study conducted earlier which explored the level of knowledge, attitude and practice (KAP) among the Rohingya refugees in Cox’s Bazar. This qualitative study was explanatory in design to understand the status of RCCE interventions and perception of the community regarding the current status of COVID-19 knowledge, attitude and practice and how these can be further improved.

Methods used in the study were (a) observation of RCCE intervention and (b) focus group discussions (FGD) with community representatives. Three clinical supervisors were selected and trained for both observation and moderation of the FGD. Five Rohingya refugee camps, selected randomly in the earlier KAP survey were selected both for the observation and FGD. CHWs are the frontline workers in the Rohingya refugee camps responsible for the RCCE activities. For observation, five CHWs were randomly selected from each of the selected five camps from the camp-wise CHW list.

The study included five Focus Group Discussions (FGD) facilitated by the Principal Investigator and Clinical supervisors, one for each camp with the view to document recommendations from the community representatives to improve compliance and practice on COVID-19 preventive measures. The participants in the FGDs were recruited from four community groups residing in five different camp locations, using a network of CHWs. Each FGD consisted of 12 representatives from the community. The selection process for study participants was purposive, at each FGD representation of a cross-section of community perspectives was ensured based on socio-demographic characteristics, economic condition, and level of vulnerabilities, including women and men, adolescents and youths, older persons, persons with disability, community leaders (*Majhi*), and religious representatives. This process ensured that the study included a diverse and representative sample of participants relevant to the research question being studied. Exclusion criteria used for the study were those having cognitive impairment or those who faced severe challenges to group participation and discussion. Table 1 showed the breakdown of different representative groups in the FGD process.

**Table 1:** Different representative groups in the FGD process.

Representative type	Men	Women
Adolescent	5	5
Youth	5	5
Older person	4	6
Person with disability	5	3
Other general community member	5	5
Community leaders	5	2
Religious representatives	5	0
Total	34	26

### Sample size

Although the sample size for qualitative research is determined by the theoretical saturation, empirical studies suggest that the recommended number of observations be between 5 and 15 and the recommended number of focus group discussions (FGDs) be between 3 and 6 with between 8 and 12 participants in each FGD (16–19). In order to conduct this study, we observed 25 CHWs and held 5 FGDs with a total of 12 participants.

### Data collection

For observation, at each of the five camps, a Clinical Supervisor was accompanied by the selected five CHWs and observed the approach of their communication, interaction between CHWs and beneficiaries, and acceptability/comprehensibility of the messages among the beneficiaries during COVID-19 risk communication. They also observed the availability and use of existing RCCE tools and materials. An open-ended observation template with a range of indicators was used for the observation, which was developed based on the recommendations from different RCCE guidelines developed by WHO and partners (20, 21).

A focus group discussion was held at each of the five camps, moderated by a Clinical Supervisor. Though the clinical supervisors were fluent in local Rohingya/Chitagongian language, a translator from local Rohingya community was engaged at each FGD to eliminate any sort of linguistic barrier between the community representatives and the moderators. Participants were invited to participate in an FGD session in a camp location familiar to them, seated in a circle or semicircle adjacent to the FGD facilitator and translator to encourage interaction and engagement. After introductions, the camp-based findings of the KAP survey conducted earlier were presented in the discussion so that the group could better understand the level of knowledge, attitude and practice and could recommend the improvement measures accordingly. Further, open-ended questions were asked using a focus group discussion guidance note (see Supplementary Annex S2) to generate discussion and comment. FGD participants were informed that there were no right or wrong answers and were encouraged to ask questions if they did not understand the session content. Average duration of an FGD was around 90 min.

The clinical supervisors were responsible for data collection both for observation and FGDs. A note taker assisted in writing up the notes as well as ensuring proper audio-recording of the discussion.

The clinical supervisors were trained on the overview of the study, the data collection instrument, and data collection procedures. They were also trained on how to obtain informed consent, how to ensure the confidentiality and privacy of the participants, and how to handle sensitive information. Both the observation template and the FGD instrument was pre-tested, respectively, among a small sample of 5 CHWs and 5 community representatives to ensure its validity and reliability. All data were stored and regularly backed up in a secure location to ensure its confidentiality and privacy. Written informed consent was obtained from all participants before the survey was administered. Participants were informed of the purpose of the study, the procedures involved, and the potential risks and benefits. They were also informed that their participation was voluntary and that they could withdraw at any time without any consequences. Overall, strict measures were taken to ensure the protection of participants’ rights and the confidentiality and privacy of their data throughout the study.

### Data analysis

The findings of the observation by the researchers were immediately noted by the clinical supervisors during the time of observation and transcribed and translated into English at the same day of the observation. The focus group discussions were recorded using audio devices and the audio recordings were transcribed verbatim (word-to-word) in English. The quality of translation was ensured by the principal investigator by comparing the translated transcript with original recording in Rohingya. Data were analyzed using a thematic analysis approach, separately for observation and focus group discussions. Software NVivo v12.0 was used to organize the data into themes. The steps of the thematic analysis included familiarizing with all the data, coding key features, grouping codes into potential themes, reviewing themes against the codes and dataset, defining and naming the themes, and writing a narrative of the analysis. To ensure the validity of the data, low relevance items (i.e., statements irrelevant to the research objectives) were eliminated, and similar concepts were merged during the analysis process.

## Ethical consideration

All respondents choose to participate voluntarily. Written informed consent was obtained from the participants for the publication of any potentially identifiable images or data included in this article. The data protection policy of the IOM is rigorously followed at every stage of the study. All recordings were temporarily stored in IOM devices and deleted after transcription. Ethical issues in the study were reviewed and approved by the Institutional Review Board of North South University (2021/OR-NSU/IRB/0401). The study adhered to the “no-harm” principle, and no intervention in the project caused significant harm to the subject population or endangered their health or lives. There was no legal risk associated with the participation of the beneficiaries in this study. Local rules/regulations were respected during interaction with the beneficiaries.

## Results

### Status and effectiveness of risk communication and community engagement approaches

The findings from the observation of community health activities have been presented in this section. Table 2 summarizes the key findings into good practices and areas needs improvement.

**Table 2:** Practices and gaps in RCCE approaches.

Good practices
Selection of CHWs from the local community
Established longstanding relationship between CHW and community
Deployment of female CHWs having better accessibility
Speaking in the local dialect
Linkage with community and religious leaders
Established trust, reliability and respect
Multiple methods of communication, posters, billboards, audio-video clips
Visualization of local customs in the promotional materials
Partially implemented good practices that need further scale-up
Use of interpersonal communication skills
Engagement with community stakeholders, e.g., community groups, Majhis, imams, TBAs
Areas need improvement
Over workload for CHWs, burdened with too many households to visit per week
Less time and effort for active listening and engagement
No guidance for CHWs on the delivery of key messages
Frequent delivery of the same messages without appropriate linkage with day-to-day practice and challenges
Messages were not adequately linked with culture, context, and resources
Less effort to link the community with appropriate resources
Cultural modes of communication (e.g., folk theaters, songs) are less explored
Less effort to empower the community/families for decision making

A CHW was assigned to cover around 150–200 households, which made them visit 20–30 households a day. They also needed to facilitate frequent community awareness-raising events, such as courtyard sessions. Most CHWs were working in the refugee camps for some years and had good familiarity with the community. Since a majority of the Rohingya men go to work in the early morning, CHWs mainly meet the female family members during their household visits. Therefore, it is more convenient for female CHWs to communicate and engage with the family members.

CHWs were either selected from the local refugee communities or from the nearby host community. Whatever their nationality, they spoke in the local dialect creating an atmosphere comfortable for the family members to communicate. Due to the long-term relationship between the CHW and the community, the family members mostly respected them and regarded them as a trusted messenger to the community. They acted as the bridge to connect the community to healthcare providers. Most often, they were well accepted and admired within the community. It was found that CHWs who worked in their catchment areas for a long duration were more engaging than those who were newly recruited. Most CHWs greeted the family members during their household visits politely. However, for some, it was found that they treated it more like routine work instead of having the greetings based on cordiality.

**Table 3:** Causes of poor compliance to COVID-19 preventive measures.

Culture, religion, and language
Religious misinterpretation and tendency to improperly replace preventive measures with religious practices
Cultural norms and perceptions hinder the practice of preventive measures
Diversity of language creates difficulty in communication and understanding
Rumor and misinterpretation of preventive messages
Local context and resources
Poverty and lack of resources affect the prioritization of COVID-19 measures, e.g., isolation, quarantine etc.
Lack of supply of preventive resources, e.g., masks, closed bins, handwashing points
Concern of security of property in case of facility-based quarantine or isolation
Physical challenges in handling with mask, e.g., interruption of communication
Congested living arrangement at the shelters
Crowding to access common water points and toilets
Trust and interaction
Breach of trust on healthcare workers due COVID-19 preventive distancing and isolation measures
Fear/rumor of isolation in case of positive COVID-19 symptoms
Inadequate compliance with preventive measures by aid workers
Communication
Limited access to reliable information; lack of community engagement events during lockdown
Inconsistency and unclarity messages; messages are not presented in a form that is understandable to the community
Improper use of channel—challenge of distancing, interruption and unclarity
Gender and social inclusion
Gender-based violence associated with isolation and treatment
Inequality of men and women in terms of use of masks and accessing services
Communication materials and messages are not responsive to the special needs of older persons and persons with disability
Stigma toward older persons, persons with disabilities, women and COVID-19 positive patients

Since they were under pressure to visit each family once a week, the CHWs could barely spend enough time with each family to engage in effective interpersonal communication. Many of the CHWs were found to spend less sufficient time listening to or addressing the concerns of beneficiaries. Some CHWs began their visit to households with a smile on their face, warm regards and engagement, however, after visiting a few households, due to lack of availability of time and exhaustion, they sometimes lost their positive body language, quickened the conversations with the family members and tried to get rid of the questions without adequate explanation.

CHWs were equipped with key messages that they need to deliver in line with the global recommendations and endorsed by local health authorities. However, the messages were not always accompanied with a guidance note on how these should be communicated with the community. In many cases, rather than providing enough context and background, many CHWs often ask some routine questions to collect information and provide routine key messages on COVID-19. CHWs were barely found providing practical examples of risk communication messages they are providing. For example, it was found that a CHW requested the household members to wash their hands and maintain respiratory hygiene and physical distance, but sometimes they did not provide any context and reason behind this measure. Also, due to the repeated delivery of the same message on every visit without any innovative method/mode, it was found many family members were hesitant to heed. There were also good examples, few CHWs were found to be able to connect with the community they were serving because they shared life experiences, listened to the community with empathy and demonstrated a deep desire to help them.

Key messages were mostly adapted from the public health preventive measures recommended globally. In some cases, messages were not enough to address the local culture, context and/or resources; and do not adequately explain how these can be achieved recognizing the limited availability of resources. For example, women were used to wearing the *Burkah* and so did not want to wear a mask underneath it; people were mostly living in very crowded location, where making physical distance maintenance difficult. The RCCE strategy and messages did not adequately address this challenge. Some CHWs were not well-oriented with the updated messages.

Most CHWs were oriented on interpersonal communication skills and tried to apply that. The weekly courtyard sessions provided a good platform for everyone to express their thoughts, fears, and concerns. The attitude of active listening varies from CHW to CHW—while some fully utilized the skill, some did not engage themselves much in active listening. Many of them used printed documents or pre-developed forms to share messages and information instead of generating interactive discussions.

Sometimes, CHWs were not provided with the information to respond to specific challenges from the community. For example, it was found challenging for CHWs to respond to how a beneficiary should access healthcare if she had three or more children and her husband was not at home, because it is difficult for her to care for three children in a health facility. Sometimes, CHWs listened to the impactful persons in the family (e.g., family head) but not to the weak or vulnerable individuals (e.g., older adult, children).

CHWs regularly received training from their agencies as well as the community health working group (CHWG). If there were any questions from the family members, CHWs tried their best to answer from their existing knowledge. However, they struggled to answer some due to lack of updated information in some cases. Some took notes of the questions so that they could communicate with their supervisor and provide the explanation on the next visit. Some CHWs were found providing information but not seeking any feedback from the community if they had any concerns or anything that needed clarification.

Some CHWs tried to explain a list of pre-identified rumor to the community, however, limited initiatives were taken by the CHWs to discover and document other rumors in the catchment area, although there was a rumor tracking system in place. The message CHW delivered focused mostly on the prevention measures, but limited attempts are taken to dispel the stigma associated with COVID-19.

For COVID-19 multiple types of communication tools were available. Flipcharts and posters were utilized by CHWs as a means of communicating risks. Sometimes, they used other forms of communication, such as radios, flyers, and audio-visual materials… It was seen that the flipcharts, posters and audio-video clips used local languages and portrayed local customs. The flip charts and posters visualized local dresses, settlements and traditions. In a few camps, especially in front of SARI ITCs, big billboards were placed by IOM with pictorials along with key messages. Through a favorite means of communication, it was found that many of the families did not have access to radios. Audio and video clips produced on COVID-19 RCCE were extensively spread among the community. However, we found that other culture-friendly media, such as folk songs and traditional theater, remained untapped for risk communication. Majhi and Imam wield considerable influence over the community, but they are typically preoccupied with other responsibilities. However, CHWs are found to involve the Majhis, imams, and traditional birth attendants, who attempted to coach community members on how to improve their health-seeking behavior and persuade them to adhere to strict preventive measures. Yet, there is scope to further enhance this collaboration between CHWs and opinion leaders to empower the community for adherence to COVID-19 preventive measures.

It was observed that most CHWs did not provide any decision-making options to the community members. They were found taking less effort to influence the family heads for taking community or family level action plans to implement the COVID-19 preventive measures. Family heads were not engaged or empowered to utilize or strengthen their leadership role in the family for monitoring and implementing the preventive measures at family level. Even if the CHWs listened to the problems of beneficiaries in terms of the inability to implement any preventive measure, no initiative was taken to link the beneficiaries to appropriate resources or stakeholders for solving the issue.

### Reasons of poor compliance to COVID-19 preventive measures and recommendations from the community

Focus group discussions were carried out with community representatives to identify the reasons behind the poor practice of COVID-19 preventive measures and generate recommendations by them to address the reasons. After analysis of the findings five key domains were identified—(a) culture, religion, and langue; (b) local context and resources; (c) community trust and interaction with aid workers; (d) method of communication; and (e) gender and social inclusion (Table 3).

#### Culture, religion, and language

##### Religious misinterpretation

The community has a strong perception that everything happens according to the will of God and what will happen cannot be altered by human. People consider COVID-19 as a disease of rich people. There is a belief that because of their religion, or because they routinely visit the mosque or pray, they are immune or protected from the illness. As opined by an Imam “*All things happen according to Allah’s will. There is nothing we can do. We will all die in this location if Allah wills it, or we will live for another year if Allah wills it.”*

People also try to correlate hygienic practices recommended for COVID-19 prevention to religious practices. For instance, there are beliefs that Hijabs can replace the use of masks for women; hand washing before prayer time (five times a day) is sufficient as hand hygiene practice. *“We do ‘Salah’ five times a day, so we do ‘wudu’ five times a day, and this is sufficient,”* explained by an Imam.

Health messages are not reflective of people’s tradition and religious beliefs. As an Imam expressed—*“NGOs have instructed us to avoid namaj in masjid, but we did not follow them. We pray in masjid regularly. Other than going to masjid Allah will never forgive us from this ‘beram’ (disease).”*

##### Cultural barriers

There is a perception in the community is that type of mask is related with different social economic status. It is expressed that *“elite people like Majhi use surgical masks and as a result those who cannot afford surgical masks are refraining from wearing any other masks for fear of social stigma.”* Sometimes, the use of masks is considered by many unsuitable in their culture. Many people think “*it is rude to talk to elders while wearing a mask*.” Some people are comfortable wearing a mask when he is alone, when they face a mentor or a senior, they consider taking off the mask as a courtesy. Many have said that maintaining physical distance with acquaintances is routinely disrespectful.

##### Language diversity

Language difference often creates difficulty in communication and understanding. Sometimes, healthcare workers are not familiar with the words of the patients, and they are unable to provide appropriate messages about the diseases to them. As expressed by one of the participants, *“doctors and nurses do not understand our language, we do not understand theirs, how will they feel our pain our problems.”* There are some audios and videos produced in Rohingya language that became popular among the community. Many IEC materials are translated into Burmese, however, Burmese is not always understandable to the community, since it’s not their mother tongue.

##### Misinterpretation, inadequate understanding

It was found among the community that there is lack of understanding of the disease, its transmission, severity and preventive measures. People often disregard the severity of the disease and try to relate it with the common cold, as expressed by one of the participants, “*We dealt with this condition frequently in our country. We were able to overcome it by using herbal medication, ginger, and warm water*.” Many people think that even if they do not wear a mask, nothing will happen to them. There is also a perception that COVID-19 is a “*disease of the rich, the poor will have nothing to do with it*.” There is belief that “*there is no corona here (in the camp), none have died*.” Some do not understand the purpose of some preventive practices, as stated by one participant, “*we do not deal with dirt, there is no reason to wash hands*!” People have negative perceptions on the SARI isolation and treatment centers, as expressed, *“The isolation centre is a “Jail” being sent to die alone, they do not give tasty food and do not allow phone calls. They confine you for days.”*

##### Recommendations from the participants

In order to improve compliance of the community to COVID-19 preventive measures, the participants expressed to address the concern of their religious belief, traditions and culture. They also urged to have health services and communication inclusive of Rohingya (in some cases Burmese) language for better understanding.

#### Local context and resources

##### Lack of resources

One of the reasons behind poor compliance is the lack of resources. Poverty causes the community to set their priority differently than COVID-19 preventive measures. As opined, “*We are poor people, many of us lost their day work in this pandemic and due to the imposed restrictions on movement, we are sometimes unable to gain access to the food distribution place. How long will we keep waiting in our house!”* An older adult participant broke out in agitation. *“We still have bloodshed still in our mind, the struggle of daily life is hard to pass by, what COVID will add on?.”* Some did not comply to facility-based isolation and quarantine measures due to lack of support to the family during their isolation/quarantine period. “*If our rations and food are not provided regularly on our house, and family is bit insecure back at home, I would not ponder overstaying there at the hospitals, I cannot sit idle knowing our house is not safe back in the downhill*” a healed COVID fighter participant stated strongly.

Participants reported that there is lack of facilities for handwashing in the camp. The supply of masks from the humanitarian agencies is also reported inadequate and infrequent. As expressed, *“eventually, we are not habituated, and you do not provide basic supplies on a regular basis. We received the cloth mask long days back and after that, there is no follow-up. No health workers coming towards our home with masks, so how they are expecting that we are going to maintain their instruction?.”* As per the opinion of community leaders, NGO services became bad during the pandemic because all washing and drain-cleaning services were discontinued. *“There is not enough bin, NGO worker told us to put cough in closed bin, not here and there but where are those? We will go to the hospital to throw it, is it?”* a female participant pointed out toward shortage of closed bin and improper waste management.

Security of shelter and resources was identified as one of the causes behind reluctance for getting isolation and treatment service. As expressed by a woman, “*when we go to your isolation centre our house, our chicken aren’t protected, we miss out our cylinder, our ration is not delivered at right time, our neighbors house was attacked by thief in the meanwhile*.”

##### Physical barriers

There are some physical factors that are limiting the practice of COVID-19 preventive measures. Some people do not comfortable wearing a mask, some find it hard to breathe when it is humid outside. Some do not practice using a mask because it interrupts communication, as expressed by one shopkeeper, “*every minute I have to talk to buyers. They do not understand me if I’m wearing a mask. And it’s hot, that’s why I do not wear a mask*.” People wearing glasses also find difficulty in wearing masks because their glass becomes blurred. For these physical factors, even if some people wear masks, they keep it under the chin. The congested living arrangement, 6–8 people in a small shelter, and extreme temperature during summer make it difficult for people to stay at their home. As raised by one representative, *“we cannot maintain social distancing in our shelter and outside as well. Our room becomes very hot during this hot summer due to the roofing materials, insufficient solar fans, and inadequate ventilation. Therefore, we cannot stay at room for long, and cannot stay outside also because of government restrictions, what you guys want us to do?”*

Some of the standard measures are not appropriate to the camp context due to the lack of physical facilities. People are living in a crowded setting and need to use common water points and toilets, which makes the practice of social distancing impossible. As stated by one of the participants, *“where they live in a house of eight to ten people, eight to ten families together use same place to use water and use a same toilet, how ‘social distance’ cannot be maintained, and what would bes the benefit of using a mask?*”

##### Recommendations from the participants

The participants urged to improve social support to the families of those who are isolated or quarantined. It should be ensured that the families are delivered with regular foods and supplies and their children are getting enough care instead of absence of one or more family members due to their stay at isolation or quarantine facility. Financial support to compensate for lost income can be considered for those staying in hospital or quarantine facility. Interventions should be explored to improve family togetherness or communication during facility-based quarantine or hospital stay.

It is expressed that regular supplies necessary for maintaining COVID-19 preventive measures should be ensured in line with the preventive messages. This includes masks, soaps, handwashing devices and a proper waste management system. There was also a request to explore adequate space at each shelter enabling home isolation/quarantine when necessary.

It was also raised to consider means of entertainment within the home environment to limit outside gathering.

- “*We are told to maintain social distance, to stay at home but how can we, our only joy come out when we got to bazar and gossip, if there was the options of television, radio we would not have the urge to go there predominantly*” one of the young ladies told.

One of the suggestions from the community to consider a basic literacy program for improving the understanding level of the community.

- “*Education will make us understand your difficult words and orders. We will be able to maintain ourselves more easily*” agreed by two of the men out there.

#### Trust and interaction

##### Breach of trust between healthcare workers and the community

Due to rumors, lockdown, and additional precautions taken by the health facilities and healthcare workers, there was mistrust between healthcare workers and community. The regular consultation service was dropped. There was fear of being killed or isolated. One of the Majhis expressed, “*We are family persons, what kind of rudeness it is not to allow us to see our family*.” Moreover, sometimes, healthcare workers and facilities were considered as the “spreaders” of the virus.

Complained by one woman, *“These days, the healthcare workers behave rudely …. If we are infected (or suspected to be) by COVID-19. The guard man and other staff got violent. If we do not have fever, they even refuse to see us after keeping us waiting for the whole day and give us only paracetamol. We locally call it ‘paracetamol center’.”*

There remains some fear among the community about the isolation centers, that is precisely due to the disbelief and mistrust. Some people believe that they will be killed, if they tested as positive for symptoms of COVID-19. However, the groups suggested due to extensive awareness-raising events, such rumors and fears have greatly reduced.

##### Inadequate compliance from aid workers

One of the factors affecting the community’s compliance to the prevention measures for COVID-19 is that humanitarian workers working in the camp are not also complying with the prevention measures, expressed by the community representatives. As expressed by a Majhi “*Why should we wear masks? Most of the law enforcement persons are not wearing, NGO workers keep gossiping closely with each other, they do not do it how can we!”*

##### Recommendations from the participants

The participants opined that the community volunteers, specially CHWs should be more engaged with the community and provide them with adequate time, attention and respect.

Also, they suggested that to develop trust and address rumors the humanitarian workers must be transparent in all their actions and communicate clearly and pro-actively with Rohingya. They should be compliant with COVID-19 preventive measures so that the community can follow them and have trust over them.

#### Communication

##### Limited access to reliable information

According to some participants, the displaced community in the camps have limited access to reliable information which made it difficult to gain knowledge and respond to the crisis. There were many rumors and misinformation, but the community had limited access to trustworthy information. Due to the initial strict lockdown during the pandemic, there was limited engagement with the community by the community volunteers or outreach teams. Household visits and community engagement events were irregular and less frequent than the normal period. There were no good alternative sources that could effectively engage the community with preventive measures.

##### Inconsistency, unclarity, and complexity of messages

There are some inconsistencies in messages due to some changes of conception on prevention. At the beginning of the COVID-19 outbreak, it was communicated that healthy people were not required to wear masks. However, later it was communicated that everyone needs to wear masks in public places to prevent COVID-19 infection. Such inconsistency of messages without explaining the reason behind the change caused confusion and affected trust.

The unclarity of messages or inadequate presentation of the content in a form that is understandable to the community was another factor of incompliance. As explained by one of the respondents, “*many of us have doubts about the rules of hand washing. Many people have misinterpreted the instructions and duration of hand washing for 20 seconds in various ways. Some have said that the government has asked them to wash their hands for 20 seconds, while others have said that they have been asked to wash their hands 20 times a day.”*

While most of the population is illiterate it is often difficult for them to interpret and understand the contents of the preventive messages. As expressed by one of the participants, *“we are the general people, we did not go to school, we cannot get complex words.”* Many of the materials and contents are not comprehendible by the general population in the camps. As expressed*, “Baba, all the things’ you people share are through mike or writing, poster with pictures are few, how much you expect us to understand through these?”*

##### Improper method of message dissemination

Although participants were familiar with different methods of message dissemination, miking through Tomtom (rickshaw-like motor vehicle) was reported as most frequently accessed source of information. However, they mentioned that such messages they cannot understand properly due to distance, interruption and unclarity. Although CHWs make frequent household visits and disseminate messages on COVID-19 prevention and control, participants mentioned that they do not stay in the house day long, so, they are not engaged with the CHWs and their messaging.

##### Recommendations from the participants

The participants suggested that folk songs or theaters can be used as one of the modes for reaching the community with risk communication messages and addressing the misinterpretation, rumors and local concerns. Facilitation, capacity building and mobilization of self-organized Rohingya groups can be considered for RCCE. They thanked the innovative approach of “go and see” visits by the community representatives to treatment and quarantine sites, which can be replicated more to enhance community trust and eradicate rumors and misconceptions. It was also suggested for more use of Burmese and Rohingya languages in promotional materials for better understanding by the community. Considering the high literacy rate, it was recommended to use meaningful culture appropriate pictures instead of text.

#### Gender and social inclusion

##### Gender

Gender inequalities impacted heavily in COVID-19 preventive measures. Women are threatened by their husbands that they will be divorced if they stay in the isolation and treatment center for treatment of themselves or their children. *“I was threatened to be sentenced ‘Talak’ by my husband if I stay in COVID center, my husband shouted at me and said there are male persons in the center who will rape me, they are bandit, characterless people.”* There is a belief that women should continue to use their “Burkah” and they do not need to wear a mask. Women are more vulnerable to stigma and social isolation if they get infected. Women are already overburdened with the pressure of taking care of family members, especially children and older adult and doing household chores. Therefore, superimposition of stigma surrounding COVID-19 causes them to hide their symptoms. One of participants opined, *“Women are not subjected to quarantine because they are responsible for their families. They (women) would be afraid of rejection from their families, particularly from their husbands.”*

##### Materials are not responsive to the special needs of vulnerable groups

The messages and the materials are not designed in a such that can be responsive to the special needs of vulnerable groups, e.g., older adult, persons with disability. One of the older adult persons stated, *“we are old folks, so we do not hear anything correctly. Most of the miking takes place on the road, far away from our house. Yeah, there are some posters here and there, but we find it tough because our vision is not great.”*

##### Stigma

There persists stigma surrounding COVID-19 in the camps. People infected with COVID-19 are stigmatized and socially isolated. Even, there is a negative perception of people toward those considered to be at higher risk of infection, such as the older adult and people with disabilities. There is a reluctance to aid or engage with those who were potentially infected or those who are considered at higher risk of infection. Also, there is fear among men that they will lose their jobs if they get infected with COVID-19. Due to fear of such stigma, people often hide their symptoms and do not present to the health facilities for isolation and treatment.

##### Recommendations from the participants

Representatives from different vulnerable groups, e.g., persons with disability and older persons requested to consider their special needs in the communication channels and messages. Some participants, specially, women also urged to address the concerns of inequalities and stigma.

## Discussion

By observing the RCCE activities, the study identified a number of effective practices, such as choosing CHWs from the local community, establishing a long-standing relationship between the community and CHWs, involving female CHWs, using the local dialect when communicating, and collaborating with community and religious leaders. We identified some effective strategies that require further expansion, including the use of multiple communication channels and instruments as well as interpersonal communication abilities. We also identified certain gaps that need to be addressed, such as CHWs being overworked with numerous tasks and household visits, less effort being put into active listening, lack of formal guidance on how to deliver key messages, reiterating the same messages without properly tying them to daily struggles, and insufficient tying of messages to culture, context, and resources. Engaging the community, including people from diverse levels and vulnerabilities, our study revealed five critical themes connected to poor COVID-19 preventive practices: (a) culture, religion, and language; (b) local context and resources; (c) community trust and interaction with aid workers; (d) communication methods; and (e) gender and social inclusion. Religious misinterpretation, cultural barriers, language diversity, misinterpretation and poor understanding, physical barriers and lack of resources required to comply with preventive measures, breach of trust between the community and aid workers, inconsistent/complex messages, lack of gender, age and social inclusion, and stigmatization are some key factors. The community recommended some measures to consider to further improve the risk communication and communication strategy. This includes addressing issues with local religious beliefs, customs, and culture, utilizing Rohingya (or, in some cases, Burmese) in communication, enhancing social support for families of isolated or quarantined patients, providing financial aid to make up for lost wages while a patient is in the hospital, fostering better family cohesion or communication during facility-based quarantine or hospital stays, ensuring a regular supply of items for maintaining COVID-19 preventive measures, creating entertainment options for the home environment to reduce outdoor gatherings, enhance the engagement of community volunteers and CHWs in the neighborhood, making an effort to increase community and humanitarian workers’ trust, exploring use of folk songs and theater for risk communication, building the capacity of Rohingya community groups, replicating the “go and see” visit strategies for treatment and quarantine sites, using culturally relevant meaningful images instead of text in communication materials, and addressing the special needs of vulnerable groups (e.g., women, persons with disability and older persons) in communication channels and messages.

A quantitative KAP study conducted by the same authors prior to this qualitative study found that the majority of the community had a good level of knowledge or awareness on COVID-19 and an average to good level of attitude, however, a significantly low level of practice toward the preventive measures (15). There was a significant improvement in knowledge and attitude among Rohingya refugees compared to the results of previous research (22), however, the practice was not improved as much as the level of knowledge and attitude (15). This improvement in the level of knowledge and attitude can be potentially linked with the extensive community outreach activities and best practices of community health interventions as identified by our study, which include the selection of CHWs from local community, deployment of female CHWs, longstanding relationship of the CHWs with the community, speaking in the local dialect, engaging with the community and religious leaders, and effort to build community trust, reliability and respect. The crucial role played by CHWs in COVID-19 RCCE were also recognized in several other studies in different settings of the world (23, 24).

The study found that overburdening the CHWs with too many tasks and a high target of coverage negatively affects their time spent per household and active listening and engagement. In Cox’s Bazar, CHWs are already assigned with the tasks of health and hygiene promotion, promotion of vector control, routine immunization, SRH awareness-raising, health referrals, defaulter tracing, community-based birth and mortality surveillance, notification of unusual events, maintaining key health and demographic data of each household and recording rumor, community complaints and providing basic first aid in the event of an emergency (25). COVID-19 RCCE and enhanced surveillance is an added responsibility to them. High workload and unrealistic expectations of work from CHWs can interfere quality of social and behavioral change communication (26). Our finding is complementary to the study of Musoke et al. (27), which highlighted that overburdening of CHWs results in stress and anxiety leading to lost working hours. It is recognized in some COVID-19 public health guidance that older people, persons with disabilities and/or chronic illnesses face higher risk of COVID-19 and face inequality and barriers to access information, education and services (28). The earlier KAP study found the association between knowledge and practice level and age group, specifically, the older adult age group (≥61 years) had less level of knowledge (AOR 0.42, *p* = 0.05) (15). This could be explained by the findings of study that the RCCE strategy, messages and the materials were not responsive to the special needs of vulnerable groups, specially, older adult and persons with disability. Our study also found the negative perception of people toward those considered to be at higher risk of infection, such as the older adult and people with disabilities. This finding is similar to Lebrasseur et al. (29) who found that the COVID-19 pandemic had a major impact on vulnerable populations, notably older people, who often experience loneliness, age discrimination, and anxiety. Therefore, the study recommends that RCCE strategies and contents should address the concerns of vulnerable groups, especially older persons and persons with disability. This is in line with the recommendation of ADCAP, which suggests identifying the barriers of older people and persons with disability, providing them access to information using a range of communication channels and different formats, using simplified languages, improving outreach strategies, and monitoring their access to ensure their effective inclusion (28). The study also discovered that gender inequality contributes to a lack of compliance with preventive measures, particularly when it comes to women who are restricted from accessing isolation and treatment services by their partners and who are more vulnerable to social isolation and stigma. However, it was also found that the RCCE strategy and contents are not adequately addressing the gender needs. Therefore, we recommend incorporating gender inclusive approaches in the RCCE strategy addressing the needs of women, men, girls and boys. This is aligned with the recommendation from a study in Pakistan, which concluded to incorporate gender aspect in designing effective communication and risk reduction strategies (30).

It is mentioned in the RCCE strategy for COVID-19 to ensure that the community engagement is culturally appropriate and empathetic (31). WHO community engagement guideline also emphasized local understanding and engagement consistent with the language, culture and context (11). Our study found that culture and religious beliefs were not adequately taken into consideration in the risk communication contents and strategies. Hence, there were religious misinterpretations and a tendency to improperly replace preventive measures with religious practices. Similarly, several cultural norms and perceptions were documented that hindered practice of preventive measures. Our study also found a lack of efforts at community health interventions in engaging the families and community in active decision making and action planning. There were initiatives to produce IEC materials, e.g., posters, key messages, in Burmese language. Some IEC materials (e.g., videos) are also produced in Rohingya language which achieved popularity (31). Since, Burmese is not the mother tongue of Rohingya and only people with some literacy can understand the language, Rohingya language should be preferred over Burmese in developing and disseminating IEC materials. Although the promotional materials well visualized local customs, the study found that there are many culture-friendly media, e.g., folk songs, traditional theater that have not yet been explored or included into the RCCE strategy. Therefore, we recommend that targeted strategies and contents should be designed to address the cultural and religious beliefs and local practices associated with COVID-19 preventive practices; and the families and community should be enabled for making informed decision and taking action to comply the preventive measure in the frame of local context. This recommendation is similar to the cultural model proposed by Airhihenbuwa et al. (32) who drew lessons from the Ebola response and HIV intervention and concluded that the COVID-19 communication strategy should be reframed to promote positive aspects of lived experience and overcome the negative practices within the context and culture of the communities. Similarly, Allgaier and Svalastog (33) also concluded that local knowledge, beliefs, and communities must be considered for effective control of Ebola outbreak with meaningful participation of local community. In many settings role of traditional and religious leaders has been well recognized including in Bangladesh, Sri Lanka and South Africa (34–36). This study also found the strong role of traditional and religious leaders, e.g., Majhis and Imams, in COVID-19 risk communication. However, this effort should be considered for further scaling up with trainings and engaging the Majhis and Imams in addressing stigma and discrimination, motivating people in testing procedures, isolation and quarantine and building community resilience.

The study also identified that insufficient resources often contribute to poor compliance. Poverty changes the community’s priorities from COVID-19 preventive efforts. Some people raised concerns on social security of the family if they remain isolated or quarantined. Security of home and resources was cited as a reason for avoiding facility-based isolation and treatment. Although preventive messages urge the use of masks, disposal into a closed waste container, and frequent handwashing, participants reported insufficiency or unavailability of some preventive tools, e.g., mask, handwashing point and closed bins. The study also identified some physical factors that limit COVID-19 preventive practices, e.g., mask causes interruption of communication and spectacle fogging. Congested living arrangements at the shelters and crowding to access common toilets and water points are also some factors that limit maintaining social distance. Our findings are contributory to Patel et al. (37), who demonstrated how people with low socio-economic status get more exposed to COVID-19 due to their poverty and several socio-economic factors, including overcrowding and unstable employment.

The study identified a breach of trust between healthcare workers and the community due to rumors, additional precautions taken by the healthcare workers, and fear regarding the isolation treatment center. Some participants also questioned the protective behavior of the healthcare workers which affected their access to the health facilities. Inadequate compliance with preventive measures by aid workers was also found as a discouraging factor for the beneficiaries to comply with the COVID-19 preventive measures. Therefore, the study recommended to take actions to strengthen trust among the community, health workers and humanitarian actors during outbreak.

## Recommendations from the study

Based on the observation of RCCE interventions, having feedback from the communities in the focus group discussions and relevant literatures and studies presented in the discussion section, the following recommendations were generated to strengthen the RCCE strategy.

### General recommendation

- Adjustments need to be made to the frequency schedule that CHWs follow when going from one household to another. This will allow the CHWs to devote sufficient time to each household, allowing them to engage in attentive listening, provide sufficient explanation, solicit feedback, and address the concerns of the residents.
- CHWs should be part of empowering families and communities to execute strategies at the family and community level to put COVID-19 preventive measures into action and monitor their effectiveness. This should be linked to the stakeholders responsible for supplying essential resources to the community, such as masks, soaps, hand washing devices, and so on.
- Consideration should be given to incorporating a variety of forms of entertainment into one’s home setting in order to reduce the need for socializing in public spaces.
- It is important to provide enhanced social support to the families of those who are hospitalized or placed in quarantine. In lieu of the absence of one or more family members as a result of their stay at an isolation or quarantine facility, it should be ensured that the families are supplied with regular foods and commodities and that their children are getting proper attention.
- People who are quarantined or hospitalized should have the option of applying for incentives to make up for lost wages while they are away from work. Efforts should be made to strengthen family cohesion and communication during hospitalization or quarantine in a facility.

### Culture, religion, and language

- Culture and context friendly methods and contents: Culture and context-friendly communication contents, methods and strategies should be designed. Different traditional methods, e.g., folk songs, theaters can be considered as further interventions.
- Extensive involvement of Majhi and Imam: Although some activities were noted regarding involvement of Majhis and Imams, these key stakeholders can be more extensively capacitated and mobilized for risk communication and behavioral change among the community.
- Use of Rohingya language: While designing information, education and communication materials attention should be given for more use of Rohingya language for better understanding by the community.
- Basic literacy program: This is one of the suggestions from the community to consider basic literacy program for improving understanding level of the community.
- Redesign risk communication contents, approaches and strategy: The risk communication messages, guidance, contents and approaches should address the following concerns as mentioned in the above section.

1. Religious belief, traditions and culture of the community should be well reflected and addressed. Any misinterpretation and misperception should be properly addressed.
2. Messages should be regularly updated based on updated scientific findings. Any confusions and unclarity of messages and guidelines should be properly explained.
3. Messages, contents, approaches and methods should address the special needs of vulnerable groups, e.g., persons with disability and older adult people.
4. Inequalities and stigma associated with COVID-19 should be well addressed in risk communication strategy.
5. Shift tone of COVID-19 messaging to a more positive message on how to support community and family members during stressful times.

The RCCE strategy should focus on bottom-up communication to reduce suspicion and improve community awareness and perception of COVID-19.

Local context and resources

- Ensure supplies: Ensure regular supplies necessary for maintaining COVID-19 preventive measures should be ensured coupled with culture and context appropriate messages. This includes, masks, soaps, handwashing devices, proper waste management system.
- Marking and signages: Placement of physical barriers and ground markings coupled with culture/language appropriate signages to maintain physical distances at crowded places, e.g., market place, distribution points etc.
- Adequate space for shelter: Consider adequate space at each shelter enabling home isolation/quarantine when necessary.

### Trust and interaction

- Addressing people’s concerns: Concerns shared day to day by the community to CHWs should be documented and shared to relevant agencies and sectors for adequately address the same.
- Interpersonal communication skill: Although the majority of CHWs receive interpersonal communication training from team trainers, the use of these skills should be effectively monitored and followed up on. Consideration can be given to providing CHWs with follow-up or refresher training. Different IPC skills, such as gentle speaking, smiling, caring, positive body language, engaging community in problem solving and decision making, active listening with attention to people’s opinion and reaction, using video or pictorial aids, analyzing the situation, taking the time to engage people, being respectful, realizing how to support and care, and creating a comfortable environment, should be well integrated into the role and approaches of CHWs.
- More engagement of the CHWs with the community: Community volunteers, specially CHWs should be more engaged with the community establishing good interpersonal communication and providing them with adequate time, attention and respect.
- Utilization of multiple channels: In order to assess the efficacy of various communication channels in Rohingya refugee camps, additional research must be conducted. Adaptation and adoption of channels should be planned as required. Instead of repeatedly using the same message and channel, inventive content and channels should be explored.
- Better compliance, cooperation and support from humanitarian workers: To develop trust and address rumors, humanitarian workers must be transparent in all their actions and communicate clearly and pro-actively with Rohingya. They should be compliant with COVID-19 preventive measures so that the community can follow them and have trust over them.
- Feedback: A well-established system should be there for getting feedback from the community should in every RCCE intervention.

### Communication

- Identify and innovate more engaging channels of communication: Proper channel of communication should be identified or innovated. For example, folk songs or theaters can be used as one of the modes for reaching the community with risk communication messages and addressing misinterpretation, rumors and local concerns. Facilitation, capacity building and mobilization of self-organized Rohingya groups can be considered for RCCE. Community representatives can be engaged in “go and see” visits to treatment and quarantine sites to enhance community trust and eradicate rumors and misconceptions.
- Addressing rumors and misinformation: A community-based surveillance system should be operated actively identify and record rumors and misinformation; based on which a response system should be established involving CHWs.
- Transparency and up-to-date information: A transparent communication system needs to be established. The CHWs should be capacitated to share their standing, updates and the possible risks or uncertainty in future. The CHWs should be provided with updated information (on situation, strategy, plans etc.) on COVID-19 by their agencies so they can share the same with the community.
- More use of pictorials: Considering high literacy rate, IEC materials should use meaningful culture appropriate pictures instead of text.
- Appropriate message: The messages should be updated addressing the existing rumors and concerns, community’s culture and context. This should be linked to access to adequate resources for effectiveness of the messages. For example, if wearing a mask is a recommendation, it should be linked how people can get a mask.
- Active listening: Active listening skills of the CHWs to be further strengthened. CHWs should establish a comfortable zone during their conversation so that the peoples’ thoughts, fears, and concerns are shared, respected and taken into account.

### Gender and social inclusion

Specific concerns and requirements of vulnerable groups, specially girls, women, persons with disability and older persons should be considered when designing the risk communication strategy and contents. The gender-based inequality and stigma should be taken into account.

## Conclusion

RCCE is the cornerstone of reducing COVID-19 transmission. The study explored the effectiveness of RCCE strategies in the Rohingya refugee camps and identified the challenges and community recommendations in relation to COVID-19 preventive measures We identified several best practices, such as recruiting CHWs from within the community, maintaining long-term relationships with CHWs, involving female CHWs, communicating in the local dialect, and establishing connections with religious leaders. We also found areas that need improvement, such as the fact that CHWs are often overworked and unable to devote sufficient time to each individual household they visit, that they often repeat the same messages without making the necessary connections to the difficulties their clients face on a daily basis, that they rarely make the effort to connect their clients’ needs with the appropriate cultural, contextual, and material resources, and that they rarely work to empower their clients and link them to those resources. Based on extensive community participation, including members of varying socioeconomic statuses and degrees of vulnerability, we identified five central themes associated with ineffective COVID-19 prevention strategies: (a) culture, religion, and language; (b) local context and resources; (c) community trust and interaction with aid workers; (d) communication methods; and (e) gender and social inclusion. Cultural barriers, limited availability of resources, distrust between the community and aid workers, inconsistent or complex messages, improper mode of message dissemination, a lack of gender and social inclusion, and stigmatization are just a few of the factors that limit to prevent the spread of disease. We encourage organization partners to use this study’s findings and recommendations to create a comprehensive risk communication and communication engagement strategy for future outbreaks that takes into account people’s culture and context, local concerns and needs, gender and social vulnerabilities.

## Data availability statement

The original contributions presented in the study are included in the article/Supplementary material, further inquiries can be directed to the corresponding author.

## Ethics statement

All respondents choose to participate voluntarily. Written informed consent was obtained from the participants for the publication of any potentially identifiable images or data included in this article. Ethical issues in the study were reviewed and approved by the Institutional Review Board of North South University (2021/OR-NSU/IRB/0401). The study adhered to the “no-harm” principle, and no intervention in the project caused significant harm to the subject population or endangered their health or lives. There was no legal risk associated with the participation of the beneficiaries in this study. Local rules/regulations were respected during interaction with the beneficiaries.

## Author contributions

CH was responsible for the conception and design of the study as well as drafting the manuscript, managed the project, and coordinated the team of researchers. All authors provided significant contributions to the project, including data collection, analysis, interpretation and revision, read and approved the final manuscript, and agreed to be accountable for all aspects of the work.

## Funding

This research was supported by the International Organization for Migration (IOM).

## Conflict of interest

The authors declare that the research was conducted in the absence of any commercial or financial relationships that could be construed as a potential conflict of interest.

## Publisher’s note

All claims expressed in this article are solely those of the authors and do not necessarily represent those of their affiliated organizations, or those of the publisher, the editors and the reviewers. Any product that may be evaluated in this article, or claim that may be made by its manufacturer, is not guaranteed or endorsed by the publisher.

## Author disclaimer

The views presented in the study are solely those of the authors and do not represent the official stance of the International Organization for Migration (IOM). This publication was issued without formal editing by IOM.

## Abbreviations

AWD: Acute watery diarrhea
CHW: Community health worker
CI: Confidence interval
CWC: Communication with community
ERC: Ethical review committee
FDMN: Forcefully displaced Myanmar nationals
FGD: Focus group discussion
IEC: Information education and communication
IOM: International organization for migration
IRB: Institutional review board
ISCG: Inter sector coordination group (ISCG)
ITC: Isolation treatment center
KAP: Knowledge, attitude, and practice
MERS-CoV: Middle east respiratory syndrome coronavirus
NSU: North South University
RCCE: Risk communication and community engagement
SARI: Severe acute respiratory infection (SARI)
SARS-CoV-2: Severe acute respiratory syndrome coronavirus 2
WASH: Water, sanitation, and hygiene
WG: Working group
WHO: World Health Organization
