# Supplementary material for: Understanding the challenges and gaps in community engagement interventions for COVID-19 prevention strategies in Rohingya refugees: a qualitative study with frontline workers and community representatives
Source: Front Public Health. 2023 Aug 3;11:1169050. doi: 10.3389/fpubh.2023.1169050 (PMC10437112; doi:10.3389/fpubh.2023.1169050)
Supplement: Supplementary file 1 [file Data_Sheet_1.docx]

**ANNEX**

**Annex 1: Observation checklist for observation of RCCE approaches:**

| 1. Trust: Has the CHW enabled to create trust with the community? Do the community feel them as a credible person – a person who can be loved and respected? Is the CHW taking any effort to create trust with the family/community during her conversation with the community? |
| --- |
| 1. Transparency and up-to-date information: How transparent the communication approach was? Is the CHW sharing her standing, current situation and the possible uncertainty in the future? How the information and messages shared by CHWs are up-to-date? Does the CHW is briefed regularly by the CHWS or their agency on COVID-19 updates and in any changes in the strategy? |
| 1. Listening: Is the CHW actively listening to the community members? Have they made any space for the community in the conversation for sharing thoughts, fears and concerns? Is there any attempt to recognize and address rumors and misinformation from the community? |
| 1. Planning: Is the CHW engaging with the community members and building their capacity so that they can make a plan for the family and community to implement the given public measures? For example, how the family will make sure they are adhering to the principle of wearing mask in public place – do they have some masks, if not how can they manage, how can they educate the family members, how the family head can monitor the compliance of other members etc. |
| 1. Addressing people’s concerns: Is there any mechanism placed to note the people’s concerns and fears; transmit it to the central planning system and address accordingly? |
| 1. Message: How consistent the message is? Is it adequately containing the information on - what is the risk, effects of the potential risk, how potential situation can be prevented, how to respond? Is the message clear, concise, consistent and call to action. |
| 1. Integrated mode: Is there any attempt to use multiple communication channels? Does the CHW refer the community to other modes of communication, e.g. posters, billboards, audios, videos, loud speakers? |
| 1. Culture/contextualization: Is the approach, methods and contents of communication cultural friendly? Are they well matched to social norms, urrent settlement, context, education background, current socio-economic status and experiences of the community? Are there some cultural and traditional methods used for risk communication (e.g. local theatre). How the peers and influential persons (e.g. Majhis) are involved and engaged in the risk communication process? |
| 1. Use of interpersonal communication skill: Does the CHW using interpersonal communication skill during her communication with community, including gentle speaking, smiling, caring, positive body language, engaging community in problem solving and decision making, active listening with attention to people’s opinion and reaction, using video or pictorial aids, analyse the situation, taking time to engage people, being respectful, realise how to support and care and making a comfortable environment. |
| 1. Comprehensibility and feedback: Is the CHW communicating in same language of the community? Can the community members understand the messages or communication from the CHW? Is the CHW asking feedback from the community members? |

**Annex 2: Foucs Group Discussion Guidance Note**

This component will also be qualitative having five focus group discussions (FGD) facilitated by the PI and Clinical supervisor (RCCE). Each FGD (at each camp selected in the early component) will have 12 representatives from Majhi, religious leaders, elderly, persons with disability, men and women to document recommendations from the community representatives to improve compliance and practice on COVID-19 preventive measures. The findings of the beneficiary survey should be presented in the discussion so that the group can better understand the level of knowledge, attitude and practice and can recommend the improvement measures accordingly. See Box 1. Focus group discussion guidance note.

| **Box 1 Focus group discussion guidance note**   1. Greet the group, introduce the team and introduce the purpose of the discussion. 2. Play an ice-break exercise to engage the members into the discussion. 3. Present an introduction to current situation of COVID-19 and what public health preventive measures should be in place to prevent or reduce the transmission. 4. Present the findings from the beneficiaries survey on the community’s knowledge, attitude and practice toward the public health measures. 5. Generate discussion on following research questions: 6. **Why there is poor knowledge/attitude/practice among the community on COVID-19 public health measures?** Explore the gaps in terms of source of information, comprehensibility of the information, acceptability among the community on the public health measures, what particular concerns are there in accepting/practicing the measures (e.g. practicing handwashing/sample providing/quarantine/isolation), what particular gaps are there for implementing the public health measures (e.g. lack of community based monitoring)? 7. **What can be done to enhance the knowledge, attitude and practice among community on COVID-19 public health measures?** Explore the possible solutions and actions based on the cause, concerns and gaps identified. Try to facilitate the group to identify actions that reflects on the community needs, concerns, contexts, culture and traditions. 8. Summarize and present the findings from the group; ask for any clarification or further explanation if needed. 9. Close the meeting by appreciating everyone’s contribution. |
| --- |
